# Male versus female inflammatory response after brain death model followed by ex vivo lung perfusion

**DOI:** 10.1186/s13293-024-00581-8

**Published:** 2024-01-29

**Authors:** Fernanda Yamamoto Ricardo-da-Silva, Roberto Armstrong-Jr, Mayara Munhoz de Assis Ramos, Marina Vidal-dos-Santos, Cristiano Jesus Correia, Petra J. Ottens, Luiz Felipe Pinho Moreira, Henri G. D. Leuvenink, Ana Cristina Breithaupt-Faloppa

**Affiliations:** 1 Laboratorio de Cirurgia Cardiovascular e Fisiopatologia da Circulação (LIM-11), HC-FMUSP, Instituto Do Coração (InCor), Faculdade de Medicina da Universidade de São Paulo, Av. Dr. Arnaldo, 455 2º Andar, Sala 2146, São Paulo, 01246-903 Brazil; 2 Department of Surgery, University Medical Centre Groningen, University of Groningen, Groningen, The Netherlands

**Keywords:** Brain death, Donor, Ex vivo lung perfusion, Lung, Rat, Sex

## Abstract

**Background:**

Ex vivo lung perfusion (EVLP) is a useful tool for assessing lung grafts quality before transplantation. Studies indicate that donor sex is as an important factor for transplant outcome, as females present higher inflammatory response to brain death (BD) than males. Here, we investigated sex differences in the lungs of rats subjected to BD followed by EVLP.

**Methods:**

Male and female Wistar rats were subjected to BD, and as controls sham animals. Arterial blood was sampled for gas analysis. Heart–lung blocks were kept in cold storage (1 h) and normothermic EVLP carried out (4 h), meanwhile ventilation parameters were recorded. Perfusate was sampled for gas analysis and IL-1β levels. Leukocyte infiltration, myeloperoxidase presence, IL-1β gene expression, and long-term release in lung culture (explant) were evaluated.

**Results:**

Brain dead females presented a low lung function after BD, compared to BD-males; however, at the end of the EVLP period oxygenation capacity decreased in all BD groups. Overall, ventilation parameters were maintained in all groups. After EVLP lung infiltrate was higher in brain dead females, with higher neutrophil content, and accompanied by high IL-1β levels, with increased gene expression and concentration in the culture medium (explant) 24 h after EVLP. Female rats presented higher lung inflammation after BD than male rats. Despite maintaining lung function and ventilation mechanics parameters for 4 h, EVLP was not able to alter this profile.

**Conclusion:**

In this context, further studies should focus on therapeutic measures to control inflammation in donor or during EVLP to increase lung quality.

## Highlights

- Ex vivo lung perfusion maintains lung function in lung grafts from brain dead rats, independently of sex;
- Inflammation is greater in female’s lung grafts even after ex vivo perfusion when compared to males.

## Plain language summary

As there is a shortage of viable lungs for transplantation, methods of lung preservation, such as ex vivo perfusion, are important. This method is a good alternative, as it will not only preserve the lungs, but also enable lung function assessment and treatment of the organs. Studies have showed that lungs from donors of the female sex have greater risk of being rejected, when transplanted to male receptors. However, it’s not certain if sex differences in anatomy, physiology and specially in immune response could interfere with the transplant result. Females do present a greater and more efficient immune response to any hazard, however after brain death this control is lost, producing a great inflammatory response as a result. Therefore, in this study we have investigated in more detail the influence of sex on the effects of brain death followed by the preservation method. Thus, we performed a brain death model in males and females rats and placed their lungs in an ex vivo lung perfusion machine. At the end of the experiment, we analyzed lung ventilation, gas exchange, and inflammatory parameters. The obtained data indicated that overall the lung ventilation and gas exchange is maintained by the ex vivo perfusion machine. Also, that lung inflammation is influenced by the sex of the donor; where the lungs from females present greater inflammation compared to the lungs from males.

## Background

The ex vivo lung perfusion (EVLP) technique was initially used as a strategy to better evaluate lung quality and function before transplantation [1]. It has also been applied to improve lungs of extended criteria or marginal donors for transplantation, specifically, normothermic EVLP has been successfully applied in clinical studies and experimental studies [2], prevented ischemia injury compared to cold preservation, while also providing similar outcomes to conventional transplants.

The lungs are one of the most vulnerable organs to brain death (BD) effects [3] and brain-dead donors are more commonly used compared to donation after circulatory death or living donation [4]. In fact, there is a low number of multi-organ brain-dead donors, and among those, only around 15%-20% of potential lungs are considered suitable for transplantation [5]. BD triggering hormonal, metabolic, hemodynamic and immune changes, as the central nervous system control will be lost. Developing as a result a systemic inflammation, that could lead to tissue injury, loss of function and preclude transplantation [6]. The autonomic storm, initiated by BD, may lead to neurogenic pulmonary edema in response to a massive increase in capillary hydrostatic pressure caused by systemic vasoconstriction. Simultaneously, the lung develops an inflammatory response by releasing inflammatory mediators and increasing the expression of adhesion molecules [6, 7].

A factor that has gained attention is the different immune responses of females and males. However, despite the increased research highlighting sex-based differences in diseases pathogenesis, pharmacokinetics and pharmacodynamics, many studies have focused on male dominated research, leading to bias research that disregard health issues related to women [8]. A study by Christie et al. [9] showed that female donors could be considered a risk factor independently associated with the development of primary graft disfunction. Afterwards, the International Society of Heart and Lung Transplantation Registry with 9,651 data on recipients, highlighted the greater risk of lung transplantation with female donors to male receptors [10]. In other long-term survival studies the combination of female donors and male recipient had poorer survival [11, 12]. Recently, Mangiameli et al. [13] reviewed articles from 1990 to 2019 and reported that matching of sex could improve lung transplant outcome, and the mismatch of female-donors and male-recipient should be avoided. Also, of notice is the study by Eberlein et al. [14] that with size-matching lungs, sex is still a factor in survival after lung transplant; with the combination female-donors and male-recipient as the worse outcome. An experimental study developed by our group [15] revealed that female rats subjected to BD have greater lung inflammation than males. This was associated with an acute reduction in the female sex hormones. Other studies have used experimental models of ischemia/reperfusion, and trauma followed by hemorrhage and have attributed lung and systemic protective effects to female sex hormones, especially estradiol [16, 17].

In this context, the immunological and physiological responses in donors of both sexes should be investigated to better understand, manage and treat the brain-dead donor accordingly in the future, possibly increasing graft viability for transplant. Additionally, the systemic events triggered by BD, cold ischemia and the impact of EVLP as a preservation tool before transplant should be considered. To this end, our study analyzed the combined effect of BD, cold ischemia and EVLP on lung inflammation and function in the lungs of male and female rats.

## Methods

### Animals

**Fig. 1:**
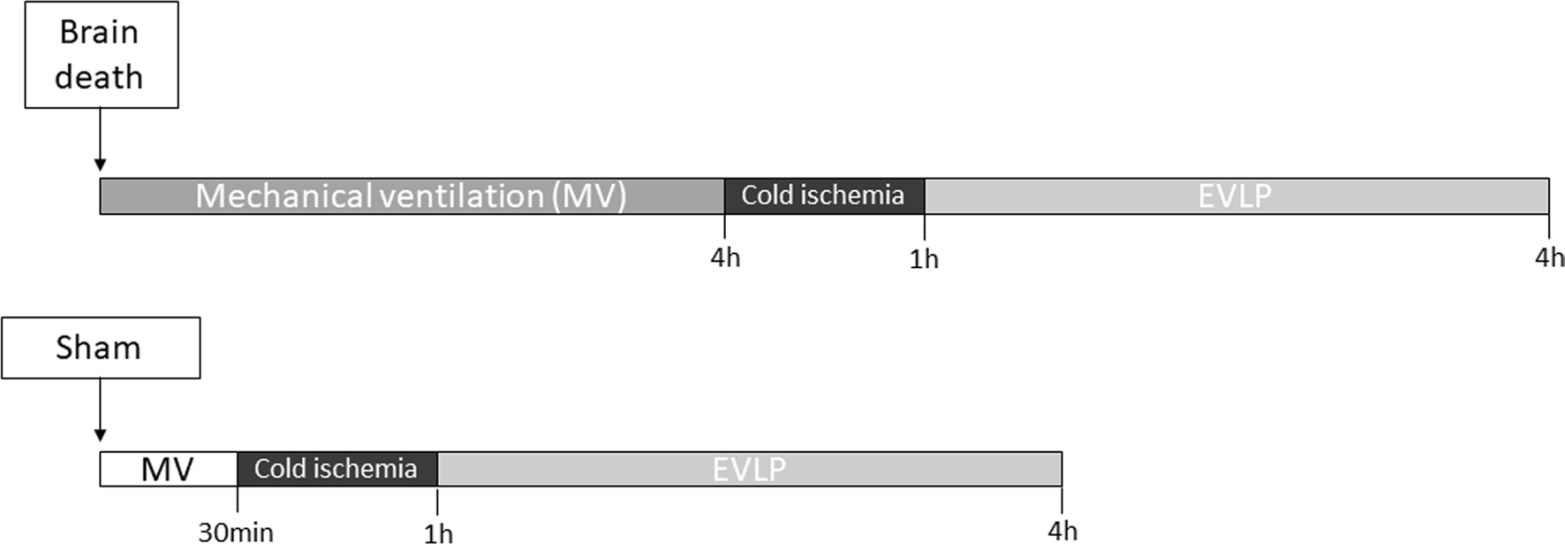
Experimental outline of the study. Female and male rats were randomly assigned to Sham or brain death (BD) group, the lungs were harvested and placed in cold ischemia followed by ex vivo lung perfusion (EVLP)

Male and female Wistar rats (8–12 weeks), obtained from Envigo (The Netherlands), were kept at 23 ± 2 °C with a 12 h light–dark cycle with free access to food and water. The animals received care in accordance with the Principles of Laboratory Animal Care (NIH Publication NO. 86–23, revised 1985) and the Dutch Law on Experimental Animal Care. This study was approved by the Institutional Animal Care and Use Committee of the University of Groningen (IACUC-RUG). The animals were divided into four groups and are illustrated in Fig. 1:

•Sham-male (*n* = 5): male rats subjected to cranial perforation and EVLP
•Sham-female (*n* = 5): female rats subjected to cranial perforation and EVLP
•BD-male (*n* = 5): male rats subjected to BD and EVLP.
•BD-female (*n* = 5): female rats subjected to BD and EVLP.

### Estrous cycle identification

In this study, we used female rats in estrus and proestrus cycle, which present higher levels of estradiol. Identification of vaginal smear cytology with optical microscope was performed in vaginal lavage with phosphate-buffered saline (PBS) and stained with crystal violet staining solution (0.2%).

### Anesthesia and BD model

Animals were anesthetized and intubated with isoflurane. A catheter was placed in the carotid artery for mean arterial pressure (MAP) measurement and in the jugular vein for fluid administration (saline solution, 2 mL/h). A catheter Fogarty-4F (Baxter Healthcare Co., USA) was inserted intracranially, and BD induced by inflation of the balloon with saline solution (≈ 30 min), a slow-induction model as described by Kolkert et al. [18]. After BD confirmation with absence of corneal reflexes and a positive apnea test, the anesthesia was interrupted and fluid administration started for the duration of the experiment (4 h). Donor management was performed as described by Kolkert et al. [18] to control MAP, body temperature, ventilation (tidal volume of 7 mL/body weight, frequency of 70 breaths/min, positive end expiratory pressure (PEEP) 1 cmH_2_O, FiO_2_ 100% for 30 min than 50%) and lung damage when MAP was > 140 mmHg. Sham animals did not have the balloon inserted and inflated and were maintained for 30 min in ventilation.

### Lung procurement and EVLP

After 4 h of BD, the lung-heart block was retrieved, the pulmonary artery was cannulated and the left ventricle excised for drainage. After flush it was preserved in cold Perfadex^®^ (XVIVO Perfusion, Sweden) for 1 h (cold storage). The normothermic EVLP method (4 h) was adapted from the study by van Zanden et al. [19]. After placing the lung-heart block in the closed system, the recruitment maneuver was performed for 3 s [15 cmH_2_O of PEEP, and 20 cmH_2_O of peak inspiratory pressure (PIP)]. Mechanical ventilation (Babylog 8000 ventilator, Draeger, Germany) was set with a tidal volume of 7 mL/kg body weight, PEEP of 5 cmH_2_O, frequency of 60 breaths/min, and fraction of inspired oxygen (FiO_2_) set at 21%. For perfusion, we used Perfadex® solution (XVIVO Perfusion, Sweden), supplemented with albumin (GE Healthcare, Austria) and augmentin (Sandoz, The Netherlands) (homemade STEEN solution). The lungs were gradually rewarmed to 37 °C (1 °C/ 2 min). Perfusion started with a pulmonary arterial pressure (PAP) of 9 mmHg, and after 10 min, maintained at 12 mmHg. The ventilation and flow parameters were continuously recorded. Samples were collected from the perfusate leaving the lung, after the stabilization period, at 15 and 240 min for analysis.

### Glucose measurement in perfusate

Perfusate samples were taken from the reservoir and the glucose content was measured with Accu-Chek Performa^®^ (Roche Diagnostics, Germany), on the time points 15, 60, 120, 180, and 240 min after EVLP started. Glucose was added (5% Baxter B.V., The Netherlands) when the levels dropped to < 9 mmol/L.

### Lung function analysis

Arterial blood was sampled from the carotid artery immediately after cannulation and 4 h after BD induction. Additionally, perfusate was collected after passing through the lung (10 min before FiO_2_ was set to 100% and gas mixture of 6% O_2_, 8% CO_2_, and 86% N_2_ turned on). Samples composition was measured using a gas analyzer machine, and parameters of partial pressure of carbon dioxide (PaCO_2_), partial pressure of oxygen (PaO_2_), and lactate were also obtained. Oxygenation status was calculated for the EVLP as PaO_2_/FiO_2_ ratio.

### IL-1β quantification

IL-1β concentration was quantified using enzyme-linked immunosorbent assay (Rat IL-1β DuoSet ELISA, R&D System, USA) in aliquots of perfusate and lung culture medium (explant) according to the manufacturer’s specifications.

### Lung histopathological analysis after ex vivo perfusion

Lung fragments were fixed in formaldehyde (10%) and embedded in paraffin. They were then sectioned (4 μm) and stained with hematoxylin/eosin. Morphological analysis was performed in each animal (*n* = 5 each group, one section per animal, five areas per section) by two blinded evaluators, analyzing parameters of leukocyte infiltration, interstitial edema, and hemorrhage. The air space/lung tissue ratio was measured using the NIS-Elements Software Basic Research (Nikon, Japan). The area analyzed for leukocyte count was 1 × 10^6^ μm^2^.

### Immunohistochemistry of lung tissue

Lung tissue paraffin Sects. (4 μm) were rehydrated and kept in ethylenediaminetetraacetic acid (EDTA, pH 8.0) antigen retrieval (15 min, 95 °C). Endogenous peroxidase was blocked (H_2_O_2_, 0.3%). Immunohistochemistry was performed using a myeloperoxidase (MPO) 1:20 primary antibody (anti-MPO, Abcam, UK), 1:100 secondary antibody (goat anti-rabbit HRP, Dako, USA), and 1:100 third antibody (rabbit anti-goat HRP, Dako). Endothelial nitric oxide synthase (eNOS) 1:50 primary antibody (anti-eNOS, Abcam, UK) and inducible nitric oxide synthase (iNOS) 1:50 primary antibody (anti-iNOS, Boster, USA), both with 1:400 secondary antibody (goat anti-rabbit HRP, Boster, USA), and 1:400 third antibody (rabbit anti-goat HRP, Santa Cruz Biotechnology, USA). Reaction was developed with 3,3’-diaminobenzidine (DAB; Thermo Fisher Scientific, USA) and counter-stained with hematoxylin. Sections were incubated in the absence of the primary antibody, as negative control. We used NIS-Elements software (Nikon) to determine the air space/lung tissue ratio and quantify stained cells. The data were expressed as the number of MPO-stained cells/mm^2^.

### Analysis of lung edema index

After the perfusion period, the superior right lung lobe was collected, weighed, and placed in an oven (100 °C, 24 h) to acquire the dry weight. Wet–dry weight ratio was used as the lung edema index.

### Gene expression analysis

Lung tissue samples were stored at − 80 °C, and the total RNA was extracted using TRIzol™ reagent (Invitrogen Life Technologies, the Netherlands) according to manufacturer’s instructions. RNA integrity was confirmed by gel electrophoresis, genomic DNA removed (Dnase I, Invitrogen Life Technologies, USA) and used for cDNA transcription (Invitrogen Life Technologies). Real-time polymerase chain reaction was performed using SYBR Green PCR Master Mix (Applied Biosystems, USA) on Taqman Applied Biosystems 7900HT RT-qPCR system (Applied Biosystems), with SYBR^®^Green primers (Applied Biosystems) against β-actin, IL-1β, IL-6, cytokine-induced neutrophil chemoattractant 1 (CINC-1), eNOS and C3 (Table 1). The amplification cycle initiated with one cycle of 50 °C for 2 min and 95 °C for 10 min, followed by 40 cycles at 60 °C for 15 s and 60 °C for 1 min. Cycle threshold (CT) values were corrected using a housekeeping gene (β-actin). For relative gene expression of IL-1 β, it was determined in relation to the Sham value (*n* = 5) of the respective sex, with no statistical difference between sexes in the Sham groups.

**Table 1:** Primer sequence used for Real-Time polymerase chain reaction analysis

Gene	Primers	Amplicon size (bp)
β-actin	5ʹ-GGAAATCGTGCGTGACATTAAA-3ʹ	74
	5ʹ-GCGGCAGTGGCCATCTC-3ʹ	
IL-1β	5ʹ-CAGCAATGGTCGGGACATAGTT-3ʹ	75
	5ʹ-GCATTAGGAATAGTGCAGCCATCT-3ʹ	
IL-6	5ʹ-CCAACTTCCAATGCTCTCCTAATG-3ʹ	89
	5ʹ-TTCAAGTGCTTTCAAGAGTTGGAT-3ʹ	
CINC-1	5ʹ-TGGTTCAGAAGATTGTCCAAAAGA-3ʹ	78
	5ʹ-ACGCCATCGGTGCAATCTA-3ʹ	
eNOS	5ʹ-AGTCCTCACCGCCTTTTCCA-3ʹ	94
	5ʹ-GCACGCGGTGAACCTCC-3ʹ	
C3	5ʹ-CAGCCTGAATGAACGACTAGACA-3ʹ	93
	5ʹ-TCAAAATCATCCGACAGCTCTATC-3ʹ	

*IL* interleukin, *CINC-1* cytokine-induced neutrophil chemoattractant 1, *eNOS* endothelial nitric oxide synthase

### Lung ex vivo culture (explant)

Lung fragments (four pieces) were incubated (37 °C, humidified atmosphere with 5% CO_2_) in culture medium (DMEM—Dulbecco’s modified Eagle medium, BioWhittaker^®^, USA), after 24 h of incubation the culture medium was collected and frozen for future analysis.

### Statistical analysis

Results are expressed as mean ± standard error of the mean (SEM) or median and 95% percentile interval (gene expression data). Data were analyzed with GraphPad Prism Software version 9 (GraphPad Software Inc., USA). Data were analyzed with two-way analysis of variance (ANOVA) followed by a two-stage linear step-up procedure of Benjamini, Krieger and Yekutieli. Data recorded over a period of time were analyzed with three-way-ANOVA corrected for multiple comparisons by controlling false discovery rate with two-stage linear step-up procedure of Benjamini, Krieger and Yekutieli. For relative gene expression of IL-1β data, we performed a Mann Whitney test.

## Results

### Lung function analysis during BD

Both groups, males and females, were subjected to BD and had arterial blood samples taken for blood gas analysis on the period before and after BD induction (Table 2). Over time, PaO_2_ decreased in both sexes, whereas PaCO_2_ was reduced in males and increased in females after 4 h of BD. Our data showed that PaCO_2_ was higher and PaO_2_ was lower in BD-female compared to BD-male.

**Table 2:** Arterial blood gas analysis before (0 h) and after 4 h of brain death induction

BD				*P* value (Time/Sex)
	Time	Male	Female	
PaCO_2_ (kPa)	0 h	9.3 ± 0.21	8.77 ± 0.5	0.6360/ 0.0193
	4 h	6.0 ± 0.57*	11.35 ± 1.4*^α^	
PaO_2_ (kPa)	0 h	53.34 ± 1.8	51.6 ± 1.8	< 0.0001/ 0.0553
	4 h	30.2 ± 3.4*	20.4 ± 1.9*^α^	

Data are expressed as the mean ± SEM from five animals. *P < 0.05, in relation to data at 0 h of the same group, ^α^*P* < 0.05 in relation to males at 4 h timepoint

**Fig. 2:**
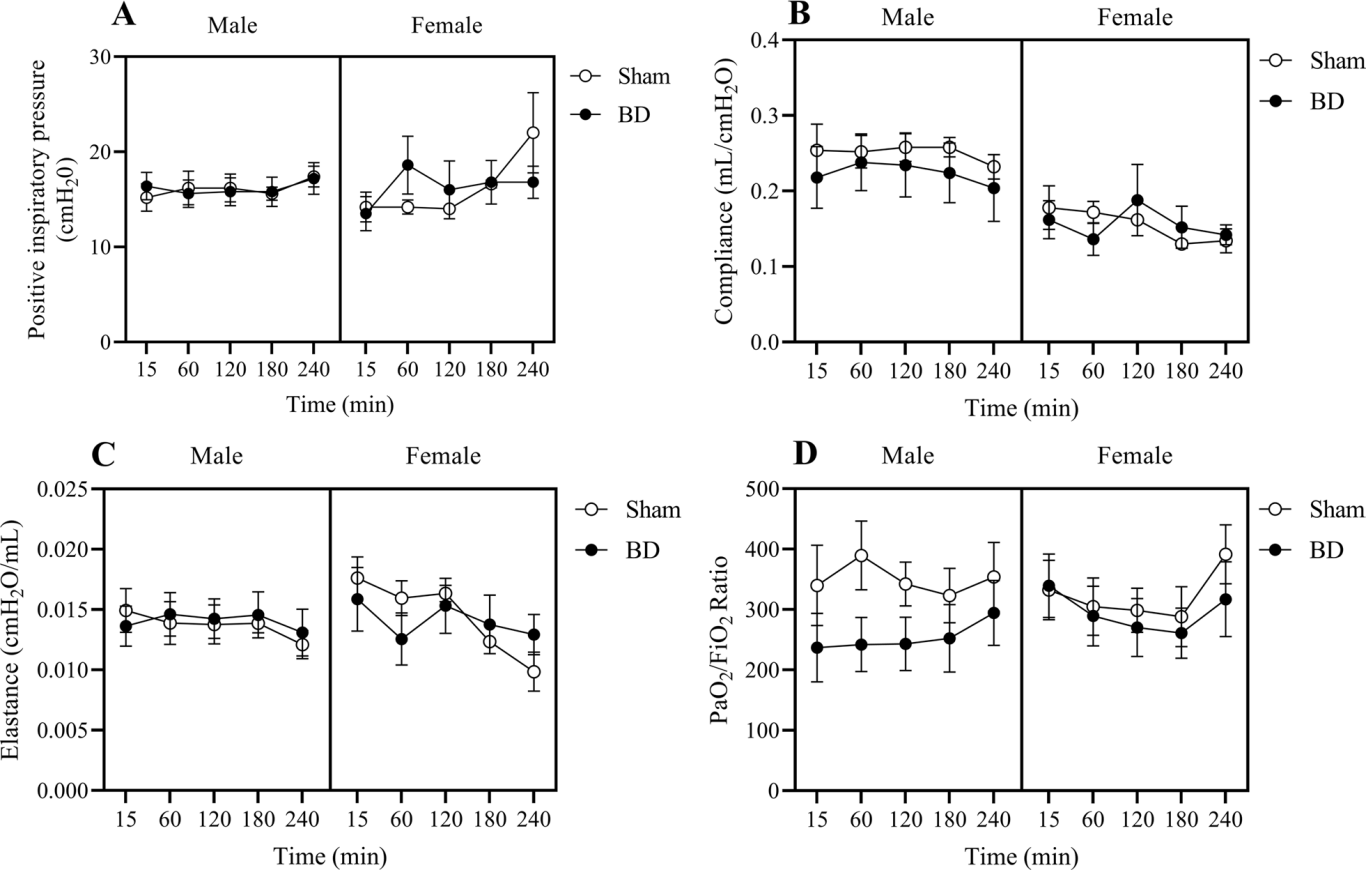
Ventilation and oxygenation performance during ex vivo lung perfusion (EVLP). Positive inspiratory pressure (**A**), compliance (**B**), elastance (**C**) and PaO_2_/FiO_2_ ratio from perfusate leaving the lung (venous) (**D**). Animals were submitted to brain death (BD—4 h) and their lungs were placed in the EVLP (4 h). As controls, Sham animals had their lungs placed in the EVLP (4 h). The data are expressed as mean ± SEM from five animals. (**B**) *P* < 0.0001 by BD; (**C**) *P* = 0.0567 by Time; (**D**) *P* = 0.0077 by Sex, calculated with three-way analysis of variance (ANOVA)

PaCO_2_ and PaO_2_ were then analyzed in the lung perfusate samples, obtained from the same rats, after 4 h of EVLP (Table 3). PaCO_2_ decreased in all groups after EVLP, while PaO_2_ was not modified by EVLP duration. Nevertheless, as shown in Fig. 2D, we observed that BD modifies the PaO_2_/FiO_2_ ratio, in which the brain-dead groups show lower values. Also, it is noticeable that BD-female presented higher PaO_2_/FiO_2_ ratio compared to BD-male. Mechanical ventilation parameters were recorded over time regarding PIP, compliance and elastance (respectively Fig. 2A–C). Compliance was affected by BD despite their sex and over time elastance was modified.

**Table 3:** Perfusate parameters on perfusate leaving the lung (venous) on ex vivo perfusion machine

	Min	Male		Female		*P* value (Time/BD/Sex)
		Sham	BD	Sham	BD	
PaCO_2_ (kPa)	15	2.02 ± 0.66	2.68 ± 0.83	2.23 ± 0.55	2.1 ± 0.33	0.0008 /0.5655 /0.1681
	240	1.64 ± 0.09	1.75 ± 0.2	1.6 ± 0.0	1.81 ± 0.48	
PaO_2_ (kPa)	15	45.32 ± 19.84	31.56 ± 16.9	44.28 ± 14.66	51.64 ± 3.44	0.6898 /0.1857 /0.2342
	240	47.18 ± 16.98	39.24 ± 15.99	52.18 ± 14.52	42.26 ± 18.47	

Animals were submitted to brain death (BD—4 h) their lungs were placed in the ex vivo lung perfusion (EVLP—4 h). As controls, Sham animals had their lungs placed in the EVLP (4 h)

Data are expressed as the mean ± SEM from five animals

### Analysis of inflammatory cells in the lungs after BD followed by EVLP

**Fig. 3:**
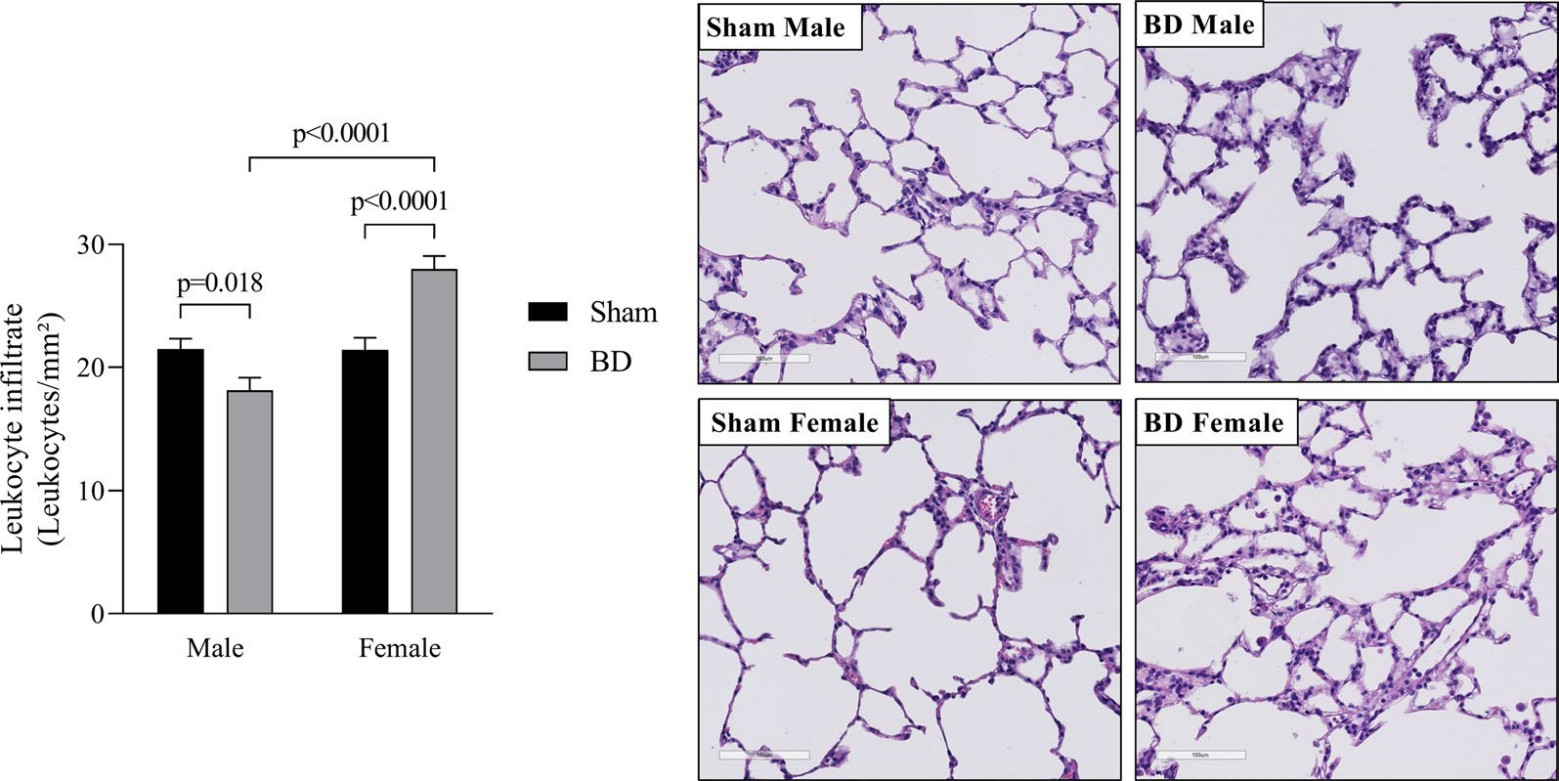
Histopathological analysis of lung tissue for lung infiltrate. Animals were submitted to brain death (BD—4 h), and their lungs were placed in the ex vivo lung perfusion (EVLP—4 h). As controls, Sham animals had their lungs placed in EVLP (4 h). Data are expressed as mean ± SEM from five animals. *P* < 0.0001 by Sex, *P* < 0.0001 by BD and *P* = 0.1114 by Sex interaction with BD, calculated with two-way analysis of variance (ANOVA)

**Fig. 4:**
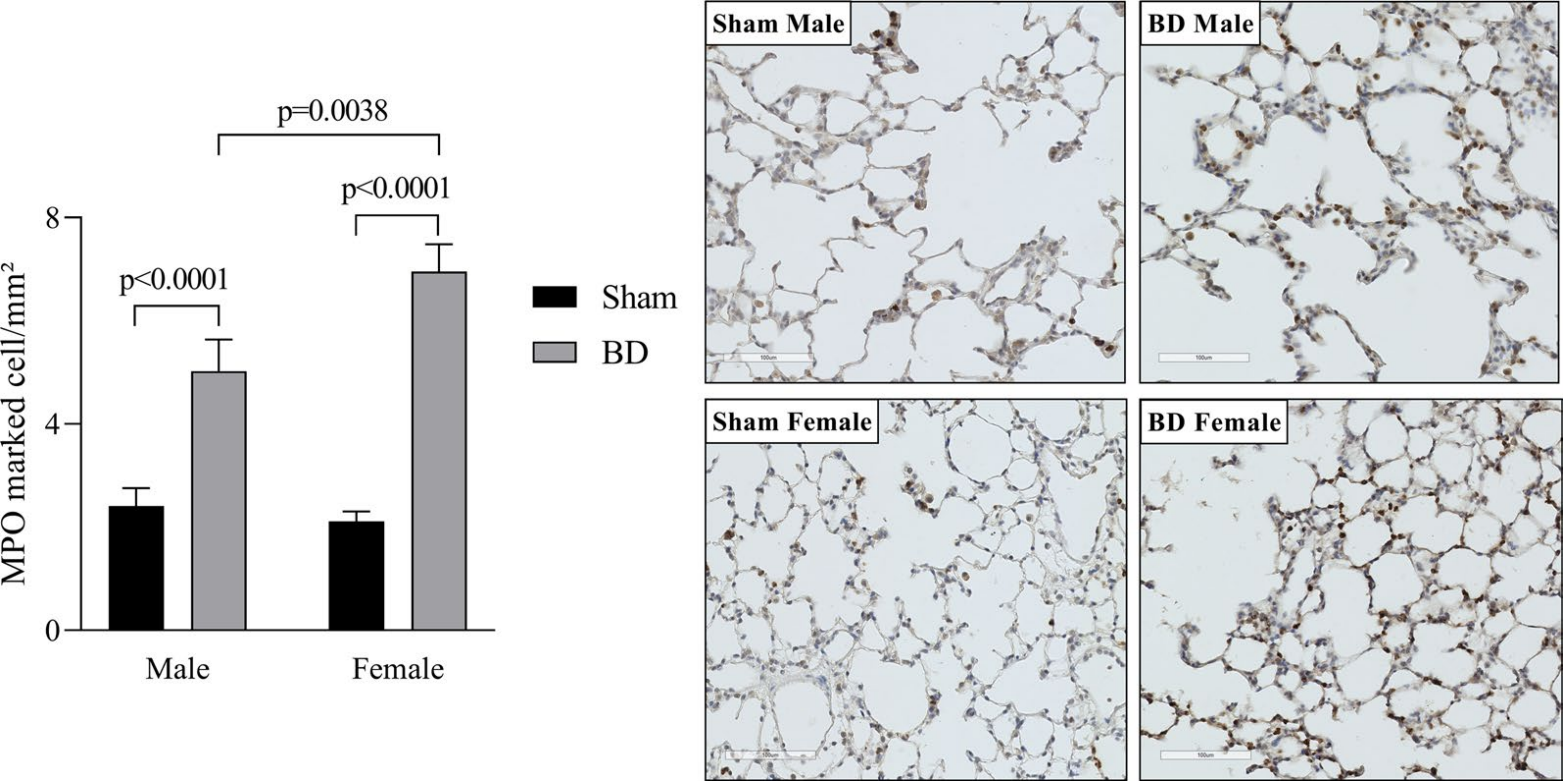
Immunohistochemistry staining for MPO marked cells in lung tissue. Animals were submitted to brain death (BD—4 h), and their lungs were placed in the ex vivo lung perfusion (EVLP—4 h). As controls, Sham animals had their lungs placed in EVLP (4 h). The data are expressed as mean ± SEM from five animals per group, two sections per animal, and five areas per section. *P* = 0.0718 by Sex, *P* < 0.0001 by BD, *P* = 0.0145 by Sex interaction with BD, calculated with two-way analysis of variance (ANOVA)

Lung tissue sections were analyzed for leukocyte infiltration (Fig. 3). Our data indicate that BD-female present a higher number of leukocytes in the lung parenchyma than brain dead males. In parallel, MPO expression was evaluated to identify the presence/activation of neutrophils in the lung (Fig. 4). Our analysis showed that BD-female had greater neutrophil infiltration than BD-male. In addition, both brain dead male and female rats showed increased MPO staining in the lung sections, compared to sex-matched Sham animals.

### Gene and protein expression of inflammatory mediators and NOS after BD followed by EVLP

**Fig. 5:**
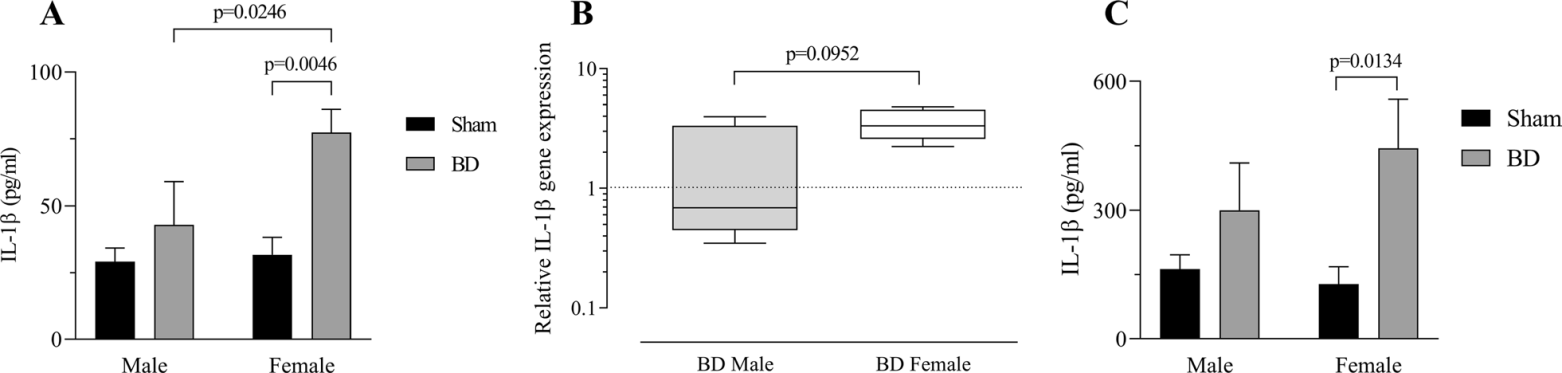
Interleukin-1β concentrations on perfusate samples at 240 min of ex vivo lung perfusion (EVLP) (**A**), gene expression (**B**), and concentration on lung explant (**C**). Animals were submitted to brain death (BD—4 h), and their lungs were placed in the EVLP (4 h). As controls, Sham animals had their lungs placed in EVLP (4 h). **A**, **C** data are expressed as mean ± SEM from five animals and **B** data is expressed as median and 95% percentile interval from five animals. **A** *P* = 0.073 by Sex, *P* = 0.007 by BD and *P* = 0.1172 by Sex interaction with BD, calculated with two-way analysis of variance (ANOVA). **B** *P* was calculated with Mann Whitney test. **C** *P* = 0.491 by Sex, *P* = 0.0093 by BD and *P* = 0.2599 Sex interaction with BD, calculated with two-way ANOVA

We also analyzed the perfusate concentrations of IL-1β after 240 min of EVLP treatment (Fig. 5A). Our data indicate that BD-female show a greater IL-1β concentration than that in BD-male. In addition, IL-1β gene was upregulated in brain dead females compared to that in brain dead males (Fig. 5B). To evaluate the long-term profile of IL-1β release, lung fragments were maintained for 24 h in culture (explant) and the released IL-1β was quantified in the medium (Fig. 5C). Its concentration was also increased in brain dead females.

**Fig. 6:**
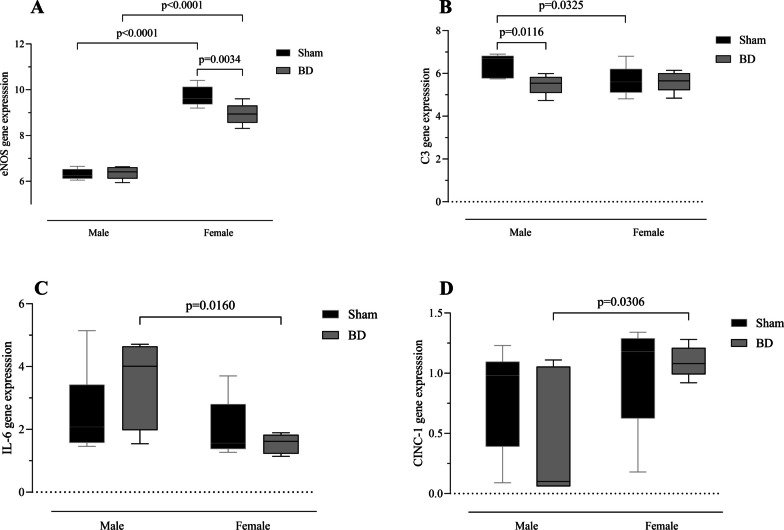
Gene expression of endothelial nitric oxide synthase (eNOS) (**A**), C3 (**B**), interleukin-6 (**C**), and cytokine-induced neutrophil chemoattractant 1 (CINC-1) (**D**). Animals were submitted to brain death (BD—4 h), and their lungs were placed in the ex vivo lung perfusion (EVLP—4 h). As controls, Sham animals had their lungs placed in EVLP (4 h). The data are expressed as median and 95% percentile interval from five animals. P was calculated with calculated with two-way ANOVA. **A** *P* < 0.0001 by Sex, *P* = 0.0384 by BD and *P* = 0.016 Sex interaction with BD. **B** *P* = 0.2045 by Sex, *P* = 0.0608 by BD and *P* = 0.0743 Sex interaction with BD. **C** *P* = 0.0233 by Sex, *P* = 0.6344 by BD and *P* = 0.1874 Sex interaction with BD. **D** *P* = 0.0387 by Sex, *P* = 0.5193 by BD and *P* = 0.2595 Sex interaction with BD

Gene expression analysis of the lung tissue indicated that eNOS was upregulated in BD-female compared to BD-male group, the same difference can be seen between Sham groups of both sexes and only on females, BD reduces eNOS expression (Fig. 6A). In C3 expression in Sham-male was higher than that Sham-female and specifically in males BD decreased their gene expression (Fig. 6B). Additionally, gene expression of IL-6 was greater in BD-male compared to BD-female group; while CINC-1 had greater expression in BD-female animals compared to BD-male (Fig. 6C, D).

**Fig. 7:**
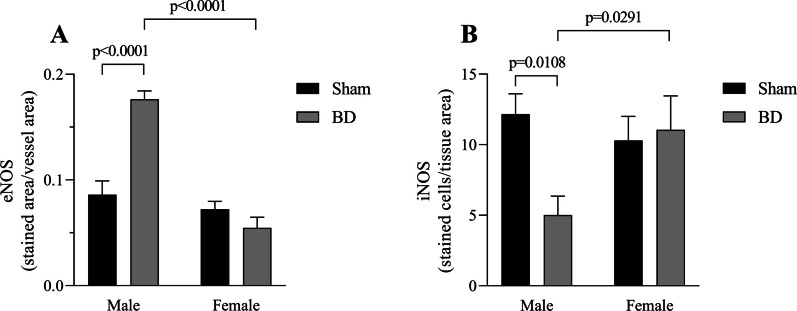
Protein expression of endothelial nitric oxide synthase (eNOS) (**A**) and induced nitric oxide synthase (iNOS) (**B**). Animals were submitted to brain death (BD—4 h), and their lungs were placed in the ex vivo lung perfusion (EVLP—4 h). As controls, Sham animals had their lungs placed in EVLP (4 h). The data are expressed as median ± SEM from five animals. **A** *P* < 0.0001 by Sex, *P* < 0.0001 by BD and *P* = 0.0013 by Sex interaction with BD, calculated with two-way analysis of variance (ANOVA). **B** *P* = 0.2604 by Sex, *P* = 0.088 by BD and *P* = 0.0371 Sex interaction with BD, calculated with two-way ANOVA

**Fig. 8:**
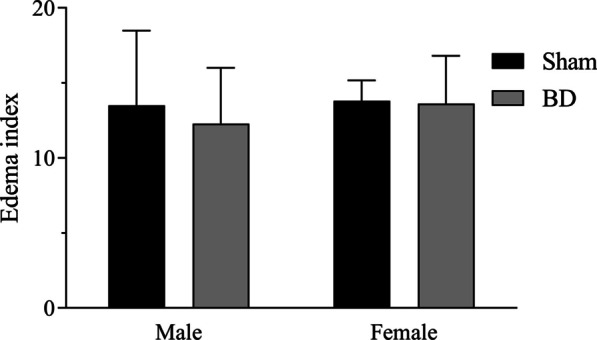
Edema index in the lungs. Animals were submitted to brain death (BD—4 h), and their lungs were placed in the ex vivo lung perfusion (EVLP—4 h). As controls, Sham animals had their lungs placed in EVLP (4 h). Data are expressed as mean ± SEM from 5–6 animals per group, two sections per animal, five areas per section. *P* = 0.6065 by Sex, *P* = 0.6558 by BD and *P* = 0.7407 by Sex interaction with BD, calculated with two-way analysis of variance (ANOVA)

Additionally, we analyzed protein expression of nitric oxide synthases (NOS) (Fig. 7), revealing that the BD-female group presents the opposite profile to the BD-male group. eNOS expression was higher in brain dead males, whereas in iNOS brain dead females had higher expression. Our analysis also showed that BD-male rats compared to their Sham inverted their response, indicating that BD followed by EVLP increased eNOS and decreased iNOS expression in the lung. Finally, when observing the edema index (Fig. 8), there was no difference among the experimental groups.

## Discussion

In the current study, we investigated sex differences in the ex vivo perfused lung grafts of brain-dead animals. This study is one of the few to evaluate lungs from animals submitted to BD, and the first to observe sex differences after machine perfusion from brain dead donors, thus providing a better translation to the clinical context. Some clinical studies have identified donor sex as a factor that could determine lung transplant success, indicating that female sex is a potential risk factor for worse transplant results [9–11]. Experimental models of BD have also shown that females, compared to males, present greater lung inflammation after BD and have associated it with the acute drop in female sex hormones [15]. Our data showed that lung function and ventilation parameters were maintained over time in all groups, with lower oxygenation capacity observed in the brain-dead groups. Most importantly, emphasizing that the higher lung inflammation produced by BD in BD-female rats, compared to BD-male, was still observed even after ex vivo perfusion, as indicated by higher leukocyte infiltrate and IL-1β release.

As described by Barklin [6], BD causes a massive release of catecholamines which will result in the hypertensive crisis and an extensive peripheral vasoconstriction, that will result in organ ischemia and metabolism shift from aerobic to anaerobic. Our study showed that lung function presented a dimorphism between sexes in the slow-induction BD model. Indeed, our analysis showed a different profile on blood gas exchange. Blood PaCO_2_ increased over time in BD-female and decreased in BD-male animals, so that BD-female presents a greater PaCO_2_ than BD-male at the end of the BD period, and blood PaO_2_ was reduced by BD in male and female animals. Patients with BD who developed acute respiratory distress syndrome (ARDS) presented an abnormally low PaO_2_/FiO_2_ ratio, with hypoxemia being the strongest independent predictor of ARDS development [20]. The main mechanisms for hypoxemia are ventilation/perfusion mismatch and shunt, which happens as a result of atelectasis and airway closure caused by lung surfactant depletion and/or increased extravascular lung fluid [21]. In this sense, it is possible to understand that brain dead female animals present worse lung function than brain dead males, corroborating data which indicate that females are more affected by BD repercussions in the lung, mainly represented by a higher inflammatory profile. In this specific study the sexual dimorphism in BD-induced inflammation was associated with the acute reduction of female sex hormones [15]. Considering the reduction of lung function in females caused by BD model, when observing the perfusate, we confirmed that oxygenation is still affected by BD, although, the sex-based differences are not observed after the grafts undergo ex vivo perfusion. Demonstrated by PaCO_2_ in perfusate samples not differing between sexes, but decreasing over time in all groups, while PaO_2_ and PaO_2_/FiO_2_ ratio were not modified by sex. However, oxygen transport to the perfusate was diminished by BD, as PaO_2_/FiO_2_ ratio was decreased in both brain-dead groups, indicating that BD did negatively affect lung function and EVLP alone did not modify that parameter. Noticing that BD-female lungs seen to take longer than 15 min to stabilize in the EVLP setting. Additionally, based on the lung mechanical ventilation data, we could conclude that EVLP maintains lung function with minor changes in mechanical ventilation.

More importantly, brain dead females showed greater lung leukocyte infiltration and higher neutrophil infiltration than brain dead male animals, which are still actively producing inflammatory mediators, proteolytic enzymes and reactive oxygen species (ROS). The same profile was reported by Breithaupt-Faloppa et al. [15] in a BD fast induction model (brain death is induced in under 1 min) with no EVLP, and it was associated with the acute reduction of female sex hormones, especially estradiol. Here we reinforce data from previous studies that showed greater systemic inflammatory profile after only BD in females [22] and indicate that this dimorphism is still present even after EVLP, as shown by this study data. Inflammatory mediators released in the perfusate, such as IL-1β, could be biomarkers of primary graft dysfunction. It has been established that the lung inflammatory response and resulting neutrophil infiltrate are important in the development of primary graft dysfunction, as the activated endothelium will promote recipient neutrophil adhesion [23, 24].

A study by Stone et al. [25] showed that acute rejection is linked to leukocyte transfer through the grafts, indicating that the depletion of leukocytes by filtration during EVLP reduces allorecognition and T cell priming. Currently, there is a controversy over the efficacy of leukocyte filters in EVLP [26]. Thus, other EVLP studies have focused on the filtration of cytokines to improve lung quality and possibly transplantation success [27]. Since a leukocyte filter was present in our circuit, we infer that leukocyte infiltration into the lung occurred during the in vivo experiment, starting after BD induction and was not modified by the perfusion period. The filter retained leukocytes that possibly adhered to the endothelial vessels without migrating into the tissue. As females present a higher inflammatory profile, with greater leukocyte infiltration future therapies should focus on their control in the donor after BD is confirmed, thus controlling leukocyte infiltration before perfusion.

In a study comparing male and female donation after circulatory death (DCD) subjected to EVLP, Mrazkova et al. [28] showed that ischemia and reperfusion (I/R) alone can modify lung perfusion and oxygenation. Females presented a higher oxygen transferability and showed lower resistance to flow, whereas males were significantly more affected by I/R injury, resulting in higher perfusion pressure. In contrast, previous studies showed that donation after brain death (DBD) is presented as a much more complex event that triggered an inflammatory process and edema formation before the I/R process [6, 7]. The present study reinforced that brain-dead patients will present lung inflammation. Females are more affected by the hormonal imbalance, accompanied by immunological and hemodynamic responses resulting from BD. One aspect that could explain this difference is the importance of female sex hormones in the inflammatory response of females [8], as after hypothalamic pituitary axis failure the body will lose control of female sex hormones and corticosterone release, affecting the inflammatory status; specially since the release of corticosterone in females under stress is linked to the release of estradiol [29]. Indeed, previous studies have investigated the contrasting responses in perfusion between sexes after BD. Mesenteric microcirculation was investigated in an acute BD model, in which the perfusion and percentage of perfused vessels were higher in females than in males. Additionally, they found that after ovariectomy, these females did not present the same response to BD. Where the maintenance of perfusion was associated with increased eNOS expression, although it may favor a greater inflammatory response in females [20]. In the lung, study by Ricardo-da-Silva et al. [30] showed that brain dead females present an increase of tissue nitrite and nitrate content, by reducing eNOS and increasing iNOS protein expression after 6 h of BD. While in males, eNOS is reduced and iNOS is increased after 3 h of BD [31]. The expression of NOS can be regulated by different factors, such as the presence of estrogens can enhance the eNOS by a unique genomic or nongenomic processes [32] or shear stress can induce iNOS expression in endothelial cells, manly through NF-κB [33]. Cytokines such as TNF-α can reduces eNOS expression, by a post-transcriptional mechanism that destabilize the mRNA molecule [34], and can also independently or synergistically with IL-1β stimulate the expression of iNOS [35].

Correia et al. [36] also established the same dimorphism during perfusion after BD, not only linking female preserved perfusion with nitric oxide (NO) synthesis, by NOS, but also possible estradiol effects on platelet activity and coagulation process. The compromised microcirculation of males during BD could be one explanation for the lower infiltration in BD-male compared to Sham-male group, impairing he recruitment of leukocytes into the lung parenchyma. Here, we observed after 4 h of EVLP, greater expression of iNOS and lower eNOS in the lungs of BD-female animals, compared to BD-male. Our data on NOS expression could reinforce the pronounced inflammatory profile of females influenced by the maintenance of microvascular perfusion that allowed leukocyte infiltration. At the same time, we observe an improvement of brain-dead male lungs as early expression of eNOS during EVLP was associated with improved allograft function [37]. On the other hand, an inhibition of iNOS has shown to prolong allograft survival in a transplant model [38].

Other studies have shown decrease of NO after ischemia and reperfusion, in both humans and animals, which could be a result of free radicals of oxygen rapid destruction [39]. Although NO in the perfusate was not investigated, other studies indicate that 3 h after BD induction females present greater NO_x_^−^ in the serum [27]. However, in the transplant model, NO bioavailability and overall activity of NOS enzymes were found to be decreased in males [40]. We suggest that in our study, EVLP could be responsible for the NOS expression change, as eNOS expression, for example, could be triggered/enhanced by stimulus such as shear-stress and 17β-estradiol [41]. In the female’s lungs, the lack of estradiol in vitro could be detrimental to the homeostasis and, as consequence, they express greater iNOS; while, in male’s lungs, the shear-stress caused by EVLP is a stimulus for eNOS expression. Further investigations of NO release could indicate the NOS enzymes’ activity and explain the dimorphism created by the combination of BD and EVLP.

Studies have proposed that the proinflammatory profile in EVLP is a consequence of tissue hypoxia, compromising cellular metabolism in lung grafts [24, 26], and releasing an array of inflammatory mediators. Andreasson et al. [24] proposed the blockage of the IL-1β pathway as a way of reducing endothelial activation and neutrophil mobilization. Our data showed that brain dead females, compared to males, released greater IL-1β levels into the perfusate, had higher gene expression, and that these differences were still identifiable 24 h after EVLP in the lung culture medium (explant). Initially, alveolar macrophages would release TNF-α and IL-1β as “early response cytokines” in the development of acute lung injury. After the transplant, IL-1β could have altered inflammatory mediators release and neutrophil infiltration [42].

It has been shown that IL-1β is formed by the cleavage of Pro-IL-1β by caspase-1, which activation in turn depends on inflammasome activity [43]. The canonical activation occurrs via pyrin domain-containing protein 3 (NLRP3) inflammasome, firstly by toll like receptors (TLR) signalling that primes the cell (converting Pro-IL-1β) and secondly by stimulus from pathogen-associated molecular patterns (PAMP) or danger-associated molecular patterns (DAMP) [44]. Sex differences observed in our study could be due to sex hormones, especially estradiol, that has been shown to inhibit NLRP3 inflammasome, caspase-1 activation and IL-1β release [45, 46]. In that sense, the reduction of estradiol, as brain dead animals present [15, 30], would increase the release of IL-1β by the lung graft. That mechanism is observed in other studies where estradiol deficiency is produced by ovariectomy or observed in menopausal women, where NLRP3/Caspase-1/ IL-1β pathway is activated [47].

In relation to complement activation, studies indicate that males generally present a greater complement system activity in basal conditions compared to females in humans [48] and mice [49]. The same is observed here, where C3 gene expression was higher in Sham-male compared to Sham-female group.

Despite bringing important information regarding sex differences in the lungs of brain-dead donors followed by EVLP, the present study had some limitations. The effects of BD alone, before cold ischemia and EVLP could not be investigated, as the study did not include a separate control group of lungs harvested after BD. There was a small number of animals per group and some of the markers analyzed were quantified based on their gene expression without the resultant protein analysis. Additionally, even considering the physiological differences between rats and humans, we haven’t followed the same time period normally necessary in the clinical setting. Future research could help to investigate these described gaps.

### Perspective and significance

In the present study we showed that during EVLP, the lungs were overall stable independently of sex, showing similar ventilation parameters by the end stage of the experiment; however, lung function was worse due to prior BD damage. More importantly, we observed sex dimorphism on lung grafts inflammatory profile, after the combined effects of BD and ex vivo perfusion. This confirms the relevance of the increased inflammation in female lung grafts, as female donor organs have been known to have worse transplant survival [9–13] and graft inflammation leads to a greater risk of rejection post-transplant [24]. Therefore, it would be of interest to develop therapies during EVLP that could target inflammation or, more effectively, control lung inflammation in the brain-dead donors, before it was harvested.

## Data Availability

The datasets used and/or analyzed during the current study are available from the corresponding author on reasonable request.
